# In-treatment behaviors in a multicomponent intervention to promote smoking cessation and prevent weight gain among smokers with obesity: A pilot study

**DOI:** 10.1192/j.eurpsy.2021.2177

**Published:** 2021-08-13

**Authors:** G. Aonso-Diego, A. Krotter, Á. García-Pérez, L. De Robles, S. Weidberg, R. Secades-Villa, G. García-Fernández

**Affiliations:** Department Of Psychology, University of Oviedo, Oviedo, Spain

**Keywords:** obesity, contingency management, weight gain prevention, smoking cessation

## Abstract

**Introduction:**

Smoking rates are quite high among overweight and obese individuals. Many smokers with excess weight are at increased risk for health complications and report that concern about post-cessation weight gain is a barrier to quitting. It is necessary to perform studies to assess the efficacy of interventions for smoking cessation among individuals with excess weight.

**Objectives:**

To describe in-treatment behaviors, in terms of smoking and weight, in an integrated intervention for smoking cessation and weight gain management.

**Methods:**

A total of 16 smokers (37.5% females, M_age_=52.31, SD=9.58) were randomly assigned to one of the two following 8-week smoking cessation conditions: 1) Cognitive-Behavioral Treatment (CBT) for gradual smoking cessation + a Weight Gain Prevention (WGP) module for weight stability (n=7); 2) the same treatment alongside Contingency Management (CM) for smoking abstinence (n=9). Smoking behavior (cigarettes per day, carbon monoxide (CO) in expired air and urine cotinine) and weight were tracked at every visit from baseline through the end of treatment.

**Results:**

Cigarettes per day significantly decreased in both conditions (p≤.028), as well as CO (p≤.018) and cotinine (p≤.043). Regarding body weight gain, participants maintained their body weight (Kg) from baseline to the end of treatment (CBT+WGP: Δ_kg_= .671, CBT+WGP+CM: Δ_kg_= .667, p≥.058) and their BMI (CBT+WGP: 30.56 vs. 30.85, CBT+WGP+CM: 29.74 vs. 29.85, p≥.139).

**Conclusions:**

Preliminary data indicated that a multicomponent intervention to promote gradual smoking cessation and prevent weight gain facilitates in-treatment tobacco reduction and weight stability. CM procedures improved in-treatment smoking behaviors.

**Disclosure:**

No significant relationships.

